# Onset of efficacy and tolerability following the initiation dosing of long-acting paliperidone palmitate: post-hoc analyses of a randomized, double-blind clinical trial

**DOI:** 10.1186/1471-244X-11-79

**Published:** 2011-05-10

**Authors:** Cynthia A Bossie, Jennifer K Sliwa, Yi-Wen Ma, Dong-Jing Fu, Larry Alphs

**Affiliations:** 1 Ortho-McNeil Janssen Scientific Affairs, LLC, Titusville, New Jersey, USA; 2 Johnson & Johnson Pharmaceutical Research & Development, LLC, Titusville, New Jersey, USA

## Abstract

**Background:**

Paliperidone palmitate is a long-acting injectable atypical antipsychotic for the acute and maintenance treatment of adults with schizophrenia. The recommended initiation dosing regimen is 234 mg on Day 1 and 156 mg on Day 8 via intramuscular (deltoid) injection; followed by 39 to 234 mg once-monthly thereafter (deltoid or gluteal). These post-hoc analyses addressed two commonly encountered clinical issues regarding the initiation dosing: the time to onset of efficacy and the associated tolerability.

**Methods:**

In a 13-week double-blind trial, 652 subjects with schizophrenia were randomized to paliperidone palmitate 39, 156, or 234 mg (corresponding to 25, 100, or 150 mg equivalents of paliperidone, respectively) or placebo (NCT#00590577). Subjects randomized to paliperidone palmitate received 234 mg on Day 1, followed by their randomized fixed dose on Day 8, and monthly thereafter, with no oral antipsychotic supplementation. The onset of efficacy was defined as the first timepoint where the paliperidone palmitate group showed significant improvement in the Positive and Negative Syndrome Scale (PANSS) score compared to placebo (Analysis of Covariance [ANCOVA] models and Last Observation Carried Forward [LOCF] methodology without adjusting for multiplicity) using data from the Days 4, 8, 22, and 36 assessments. Adverse event (AE) rates and relative risks (RR) with 95% confidence intervals (CI) versus placebo were determined.

**Results:**

Paliperidone palmitate 234 mg on Day 1 was associated with greater improvement than placebo on Least Squares (LS) mean PANSS total score at Day 8 (p = 0.037). After the Day 8 injection of 156 mg, there was continued PANSS improvement at Day 22 (p ≤ 0.007 vs. placebo) and Day 36 (p < 0.001). Taken together with results in the 39 mg and 234 mg Day 8 arms, these findings suggest a trend towards a dose-dependent response. During Days 1 to 7, AEs reported in ≥2% of paliperidone palmitate subjects (234 mg) and a greater proportion of paliperidone palmitate than placebo subjects were: agitation (3.2% vs. 1.3%; RR 2.52 [95% CI 0.583, 10.904]), headache (4.0% vs. 3.8%; RR 1.06 [95% CI 0.433, 2.619]), and injection site pain (6.7% vs. 3.8%; RR 1.79 [95% CI 0.764, 4.208]). Days 8 to 36 AEs meeting the same criteria in the 156 mg Day 8 arm were: anxiety (3.1% vs. 2.5%; RR 1.24 [95% CI 0.340, 4.542]), psychotic disorder (2.5% vs. 1.3%; RR 1.99 [95% CI 0.369, 10.699]), dizziness (2.5% vs. 1.3%; RR 1.99 [95% CI 0.369, 10.699]), and injection site pain (2.5% vs. 1.3%; RR 1.99 [95% CI 0.369, 10.699]). Corresponding Days 8 to 36 AEs in the 39 mg Day 8 group were: agitation (4.5% vs. 4.4%; RR 1.03 [95% CI 0.371, 2.874]), anxiety (3.9% vs. 2.5%; RR 1.55 [95% CI 0.446, 5.381]), and psychotic disorder (2.6% vs. 1.3%; RR 2.07 [95% CI 0.384, 11.110]) while in the 234 mg Day 8 group it was anxiety (3.1% vs. 2.5%, RR 1.25 [95% CI 0.342, 4.570]).

**Conclusions:**

Significantly greater symptom improvement was observed by Day 8 with paliperidone palmitate (234 mg on Day 1) compared to placebo; this effect was maintained after the 156 mg Day 8 injection, with a trend towards a dose-dependent response. No unexpected tolerability findings were noted in the first week or month after the initiation dosing.

**Trial registration:**

ClinicalTrials.gov: NCT#00590577

## Background

For individuals with schizophrenia--whether a first episode or a relapse--the rapid and robust control of symptoms at well tolerated medication dosages are primary goals to reduce emotional distress, minimize disruption to the patient's life, and reduce the risk of dangerous behaviors [1]. Evidence also suggests that the prompt improvement in symptoms may improve long-term outcomes [2]. To realize these benefits, the effectiveness of a therapeutic agent measured as an improvement in symptoms, acceptable tolerability, and an early onset of effect are important considerations in the choice of an antipsychotic agent.

Rapid symptom control is a strong predictor of treatment success and may be a valuable indicator of long-term symptom control as well as a low rate of relapse and rehospitalization, which may contribute to reducing healthcare costs [3,4]. While some data suggest that most symptom amelioration occurs within the first 2 weeks after introduction of antipsychotic treatment, some patients require a longer time to respond. In a recently published investigation of patterns of response (defined as ≥30% reduction in Positive and Negative Symptom Score [PANSS] from baseline) with an atypical antipsychotic, approximately 36% of patients responded within 2 weeks, while an additional 20% responded by week 6 [5]. The American Psychiatric Association guidelines recommend a 2- to 4-week therapeutic trial prior to changing a treatment regimen [1].

Paliperidone palmitate is a long-acting injectable formulation of paliperidone, which is also formulated for daily oral administration as paliperidone extended-release (ER). Paliperidone palmitate is the palmitate ester of paliperidone. The dosing of paliperidone palmitate may be expressed in terms of milligrams (mg) of paliperidone palmitate or in terms of milligram equivalents (mg eq) of the pharmacologically active fraction, paliperidone. Paliperidone palmitate expressed as 39, 156, and 234 mg is equivalent to 25, 100, and 150 mg eq, respectively, of the active fraction paliperidone. The pharmacokinetic properties of paliperidone palmitate allow for once-monthly injections following two initiation doses given 1 week apart [6-8]. Pharmacokinetic data indicate higher median peak concentrations following paliperidone palmitate administration into the deltoid rather than the gluteal muscle, with similar area-under-the-curve (AUC) values [7]. Given this, it is recommended that administration of initiation doses of paliperidone palmitate be in the deltoid muscle with maintenance dose administration being interchangeable between deltoid and gluteal administration [7].

Paliperidone palmitate has been studied in several randomized, double-blind controlled trials using various dosing regimens [9-14]. A recently completed phase 3 trial was the first placebo-controlled study to assess paliperidone palmitate administered at the recommended Day 1 dose of 234 mg by deltoid injection. Subjects then received 39, 156, or 234 mg on Day 8 and monthly thereafter (deltoid or gluteal). In this study, paliperidone palmitate, without oral antipsychotic supplementation, was associated with significant improvements in symptomatology with no unexpected tolerability findings in adults with symptomatic schizophrenia, at all doses tested [14].

An early, well-tolerated response to antipsychotic treatment has important down-stream implications for long-term symptom control, treatment adherence, healthcare costs, and, consequently, clinical decision-making. These post-hoc analyses of data from the published trial [14] was designed to address two commonly encountered clinical questions associated with the initiation regimen of paliperidone palmitate: 1) when is the onset of efficacy and; 2) how well is this initiation dose tolerated. This report focuses on the subjects who received 234 mg on Day 1 (deltoid) followed by 156 mg on Day 8 (deltoid or gluteal). Data are also presented for those who received 234 mg on Day 1 followed by 39 or 234 mg on Day 8.

## Methods

### Design

A 13-week double-blind, randomized, placebo-controlled phase 3 trial (NCT#00590577) was conducted from March 2007 to March 2008 at 72 centers in 8 countries in North America, Europe, and Asia. Subjects with schizophrenia and a PANSS total score of 70 to 120 (inclusive) at screening and 60 to 120 (inclusive) at double-blind baseline were eligible for study enrollment. Key exclusion criteria included primary DSM-IV Axis I diagnosis other than schizophrenia, DSM-IV diagnosis of active substance dependence within 3 months before screening, history of treatment resistance (failure to respond to 2 adequate courses of different antipsychotic medications with a minimum of 4 weeks duration at the patient's maximum tolerated dose), history of neuroleptic malignant syndrome, a relevant history of any significant or unstable systemic disease, morbid obesity (body mass index ≥40 kg/m^2^), and circumstances that could increase the risk of the occurrence of Torsade de Pointes or sudden death. Further details of the study design are reported by Pandina et al. [14].

### Study Medications

The study consisted of a screening period of up to 7 days to washout disallowed psychotropic medications followed by a 13-week double-blind treatment period. On Day 1, eligible patients were randomly assigned (1:1:1:1) to fixed doses of paliperidone palmitate 39, 156, or 234 mg (equivalent to 25, 100, or 150 mg eq of the active fraction paliperidone), or placebo, based on a computer-generated randomization schedule balanced by using permuted blocks of treatments and stratified by center. On Day 1, all patients received a deltoid injection of paliperidone palmitate 234 mg or matching placebo. On Day 8, and then on Days 36 and 64, patients received their assigned treatment per the randomization schedule, injected in the deltoid or the gluteal muscle at the discretion of the investigator. Patients were hospitalized from Day 1 (first injection) until at least after the second injection of study drug on Day 8. Antipsychotics except study drug were prohibited during the double-blind treatment period. Prior antiparkinsonian medications were to be washed out prior to baseline, but were allowed during the study at the discretion of the investigator if extrapyramidal symptoms [EPS] emerged or worsened. Oral benzodiazepines were allowed for agitation, anxiety, or sleep difficulties at the permitted maximum daily doses.

### Assessments

Efficacy was assessed using PANSS total scores at Days 4, 8, 22, 36, 64, and 92 (or study endpoint). Tolerability assessments included treatment-emergent adverse events reports and adverse-event related study discontinuations.

### Analysis Sets and Statistical Evaluations

Onset of efficacy and tolerability analyses were performed on the intent-to-treat (ITT) analysis set, which included all randomized patients who received at least one dose of double-blind study medication and had both the baseline and at least one post baseline efficacy assessment. Changes from baseline in PANSS total scores were estimated by Least Squares (LS) means and compared between groups using an Analysis of Covariance (ANCOVA) model and Last Observation Carried Forward (LOCF) methodology, without adjustment for multiplicity. Results on Days 4 and 8 were pooled for the paliperidone palmitate dose arms (all received 234 mg of paliperidone palmitate on Day 1). At Days 22 and 36, data were analyzed for each paliperidone palmitate dose group (corresponding to 39, 156, or 234 mg that was administered at Day 8). The effect size (Cohen's d) for paliperidone palmitate relative to placebo in PANSS change from baseline was calculated using Cohen's d with LS means and mean (standard error [SE]) from ANCOVA model for treatment comparison. Onset of efficacy was defined as the first timepoint at which the change from baseline in the PANSS total score in the paliperidone palmitate group was significant compared to placebo (at the 2-sided nominal 5% level of significance). Responder rates were defined as the proportion of subjects with a ≥30% reduction from baseline in PANSS total score. Pairwise comparisons were performed using Cochran-Mantel-Haenszel tests controlling for country.

Adverse events that occurred in ≥2% of paliperidone palmitate subjects were summarized (by dose arm) and compared to placebo, with determination of the relative risk (RR) and 95% confidence interval (95% CI) associated with paliperidone palmitate. RRs were considered statistically significant when 95% CIs did not include 1. No adjustments were made for multiplicity. Tolerability associated with the initiation dosing regimen was also assessed through analyses of treatment discontinuation and adverse event reports (including extrapyramidal-, metabolic- and potentially prolactin-related events) during post-injection time periods of Days 1 to 7 and 8 to 36. Extrapyramidal events included akathisia, tremor, dyskinesia, extrapyramidal disorder, movement disorder, or parkinsonism. Metabolic events included metabolism or nutritional disorders such as increased/decreased appetite, increased/decreased weight, dyslipidemia(s), or malnutrition. Potentially prolactin-related events included reproductive system events, breast disorders, and ejaculation disorders.

## Results

### Patient Disposition and Characteristics

**Figure 1:**
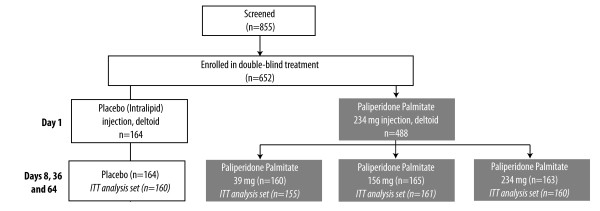
**Subject Randomization**. Of 652 subjects enrolled in the double-blind treatment period, 488 were randomized (1:1:1:1) to paliperidone palmitate (fixed dose of 39, 156, or 234 mg) and 164 to placebo. All those randomized to the fixed doses of paliperidone palmitate received 234 mg as the first initiation dose on Day 1, followed by administration of their fixed dose on Days 8, 36, and 64.

Of 855 subjects screened, 652 (76%) were randomized to either paliperidone palmitate (n = 488) or placebo (n = 164); 476 and 160, respectively, were in the ITT analysis set (Figure 1). All ITT subjects randomized to paliperidone palmitate received a Day 1 dose of 234 mg; 161 were randomized to the 156 mg Day 8 treatment arm. One-hundred sixty (160) and 155 subjects were randomized to the 234 and 39 mg Day 8 treatment arms, respectively. Administration of the Day 1 doses were primarily (99%) in the deltoid muscle (3 paliperidone palmitate and 1 placebo subject received the injection in the gluteus). The Day 8 dose was administered in the gluteus in 48% to 53% of those in the paliperidone palmitate treatment arms and in 58% of those in the placebo treatment arm.

Baseline demographics and disease characteristics in the ITT analysis set were similar across treatment arms with a mean age of 39 years, 67% male, and 54% Caucasian [14]. Mean (Standard Deviation [SD]) PANSS total score scores were 86.8 (10.31) in the placebo group and 86.9 (11.99), 86.2 (10.77), and 88.4 (11.70) in the paliperidone palmitate 39, 156, and 234 mg Day 8 arms, respectively. Atypical antipsychotics were commonly used (70% of subjects) prior to enrollment, with oral risperidone use reported by 34% to 41% of subjects across the arms. Prior to baseline, approximately 30% of subjects in each treatment arm were using an anti-EPS medication (24% placebo and 35%, 30% and 33% in the paliperidone palmitate 39, 156, and 234 mg Day 8 arms, respectively) and approximately 60% were using a benzodiazepine (65%, 67%, 58%, and 59%, respectively).

### Effects on PANSS Total Scores

#### At Day 8 Timepoint

**Figure 2:**
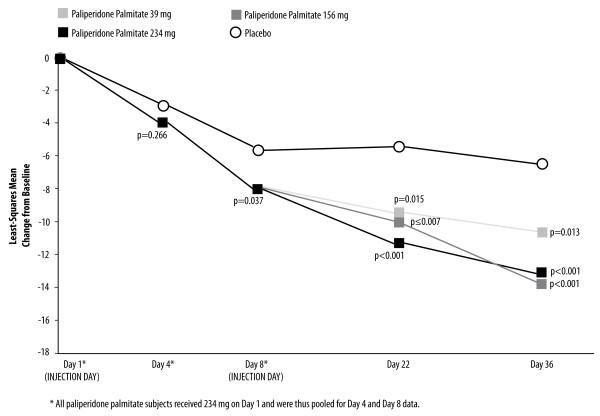
**Changes in PANSS Total Scores Over Time (LOCF) in the ITT Analysis Set (p-values for Paliperidone Palmitate vs. Placebo)**. The administration of paliperidone palmitate 234 mg on Day 1 was associated with a significantly greater improvement than placebo on mean PANSS total score at the Day 8 assessment (LS mean [SE] change from baseline -8.21 [0.87] vs. -5.79 [1.20], p = 0.037). In a dose-dependent fashion, all paliperidone palmitate groups continued to show greater PANSS total score improvement than placebo at subsequent timepoints.

Paliperidone palmitate 234 mg administered on Day 1 was associated with a significantly greater improvement than placebo on mean PANSS total score at the Day 8 assessment (LS mean [SE] change from baseline -8.21 [0.87] vs. -5.79 [1.20], p = 0.037) (Figure 2). The placebo vs. treatment effect size (95% CI) was 0.19 (0.01, 0.37) (Table 1).

**Table 1:** Effect size for PANSS total change score: Paliperidone palmitate vs. placebo (95% CI)

Paliperidone Palmitate Treatment Group				
	**234 mg (n = 459-Day 4; n = 476-Day 8)**	**39 mg (n = 155)**	**156 mg (n = 161)**	**234 mg (n = 160)**

Day 4*	0.10 (-0.08, 0.29)			

Day 8*	0.19 (0.01, 0.37)			

Day 22		0.27 (0.05, 0.50)	0.30 (0.08, 0.52)	0.41 (0.19, 0.63)

Day 36		0.28 (0.06, 0.50)	0.43 (0.21, 0.64)	0.40 (0.18, 0.62)

*All paliperidone palmitate dose groups received 234 mg on Day 1, and their assigned dose on Day 8.

Type of effect size is Cohen's d; p-value is from two-sided Z test.

#### At Day 22 and Day 36 Timepoints

After the Day 8 injection of 39, 156, or 234 mg, all paliperidone palmitate groups continued to show greater PANSS total score improvement than placebo at the subsequent Days 22 and 36 timepoints (Figure 2). Among those administered the recommended 156 mg Day 8 dose of paliperidone palmitate vs. placebo, the Day 22 LS mean (SE) change from baseline was -9.9 (1.38) vs. -5.4 (1.4), p ≤ 0.007, with further improvement at Day 36 (LS mean [SE] change from baseline: -13.2 [1.48] vs. -6.5 [1.50], p < 0.001). Corresponding effect sizes for all dose arms are shown in Table 1. These results suggest a dose-related effect.

**Figure 3:**
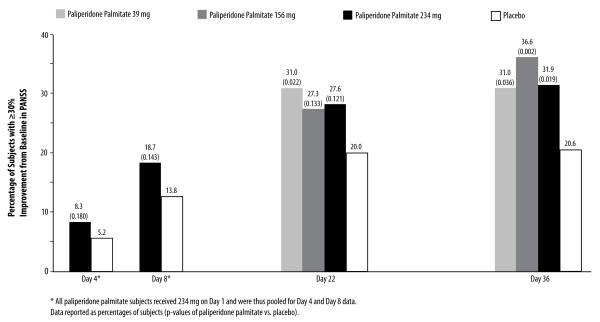
**Responders: ≥30% Improvement from Baseline in PANSS Total Scores**. The responder rates were significantly higher with paliperidone palmitate (all dose groups) than with placebo by the Day 36 timepoint.

The responder rates (≥30% reduction from baseline in PANSS total score) were significantly higher with paliperidone palmitate (all dose groups) than with placebo by the Day 36 timepoint (Figure 3). In the group receiving the 156 mg Day 8 dose, the responder rate was 36.6% compared to 20.6% with placebo (p = 0.002) (Figure 3).

### Discontinuations and Benzodiazepine Use

#### Days 1 to 7

During Days 1 to 7, the percentage of patients who discontinued study participation was 2.9% in those who received paliperidone palmitate (234 mg Day 1) and 4.4% in the placebo group (Table 2). During the week following the first injection, the most common reason for discontinuation in both groups was withdrawal of consent (1.9% in placebo and 1.1% in paliperidone palmitate).

**Table 2:** Study Discontinuations and Benzodiazepine Use, by Treatment Group and Time Period

	**Days 1 to 7**		**Days 8 to 36**			
	
	**Placebo (n = 160)**	**Paliperidone palmitate 234 mg ** **(n = 476)**	**Placebo (n = 160)**	**Paliperidone palmitate 39 mg ** **(n = 155)**	**Paliperidone palmitate 156 mg ** **(n = 161)**	**Paliperidone palmitate 234 mg ** **(n = 160)**
	
**Discontinuations, No., (%)**	7 (4.4%)	14 (2.9%)	52 (32.5%)	38 (24.5%)	38 (23.6%)	32 (20.0%)
	
**Discontinuation Reason, No., (%)**						
	
*Lack of efficacy*	2 (1.3%)	3 (0.6%)	29 (18.1%)	14 (9.0%)	14 (8.7%)	16 (10.0%)
	
*Withdrawal of consent*	3 (1.9%)	5 (1.1%)	12 (7.5%)	12 (7.7%)	17 (10.6%)	10 (6.3%)
	
*Adverse event*	2 (1.3%)	4 (0.8%)	5 (3.1%)	8 (5.2%)	5 (3.1%)	5 (3.1%)
	
*Lost to follow-up*	0	0	6 (3.8%)	4 (2.6%)	1 (0.6%)	1 (0.6%)
	
*Other*	0	2(0.4%)	0	0	1 (0.6%)	0
	
**Benzodiazepine Use, No. (%)**	80 (50%)	247 (51.9%)	70 (43.8%)	66 (42.6%)	54 (33.5%)	64 (40.0%)

*All paliperidone palmitate dose groups received 234 mg on Day 1, and their assigned dose on Day 8.

Withdrawal due to adverse events was low in both groups (0.8% [n = 4] in paliperidone palmitate and 1.3% [n = 2] in placebo). Events that resulted in discontinuation in the paliperidone palmitate group were: gastroesophageal reflux, pain in extremity, suicidal ideation, and toothache (1 subject); injection site pain (1 subject); insomnia, schizophrenia, and tremor (1 subject); and psychotic disorder (1 subject). Adverse events leading to discontinuation in the two placebo-treated subjects were schizophrenia (1 subject) and schizophrenia, increased aspartate aminotransferase and increased blood lactate dehydrogenase (1 subject).

Approximately half the patients in both groups reported benzodiazepine use (Table 2).

#### Days 8 to 36

In the month following the Day 8 injection (Days 8 to 36), discontinuation rates were 32.5% in the placebo group and 23.6% in the paliperidone palmitate 156 mg Day 8 group (Table 2). Discontinuation due to adverse events was 3.1% (n = 5 in each group) in the placebo group as well as the paliperidone palmitate 156 mg Day 8 treatment arm. Adverse events leading to discontinuation in the 5 placebo-treated subjects were: increased alanine aminotransferase and increased aspartate aminotransferase (1 subject); nausea and vomiting (1 subject); delusional disorder-persecutory type, musculoskeletal stiffness, and tremor (1 subject); anxiety and schizophrenia (1 subject); and insomnia and schizophrenia (1 subject). Events leading to discontinuation in the paliperidone palmitate 156 mg Day 8 group were: schizophrenia (2 subjects); schizophrenia-paranoid type (1 subject); insomnia, otitis media-chronic, psychotic disorder, and toothache (1 subject); and injection site swelling (1 subject).

No subject discontinued due to adverse events in the paliperidone palmitate 39 mg Day 8 arm; 5 discontinued in the 234 mg Day 8 treatment arm. Adverse events in the latter group were psychiatric disorder and toothache (1 subject); anxiety (1 subject); agitation, aspartate aminotransferase increase, toothache, and white blood cell count decrease (1 subject); agitation, insomnia, and schizophrenia (1 subject); and blood amylase increased and cerebrovascular accident (1 subject).

Benzodiazepine use during this period was reported by 43.8% of the placebo arm and 33.5% of the paliperidone palmitate 156 mg Day 8 arm. Rates were 42.6% in the 39 mg Day 8 arm and 40.0% in the 234 mg Day 8 arm (Table 2).

### Adverse Events

#### Days 1 to 7

The overall rate of adverse events during the week following the paliperidone palmitate 234 mg Day 1 initiation dose was similar to that seen with placebo (38.0% [181/476] vs. 43.1% [69/160], respectively). With the exception of one report of schizophrenia in a placebo-treated subject, no other adverse events were rated as serious.

**Figure 4:**
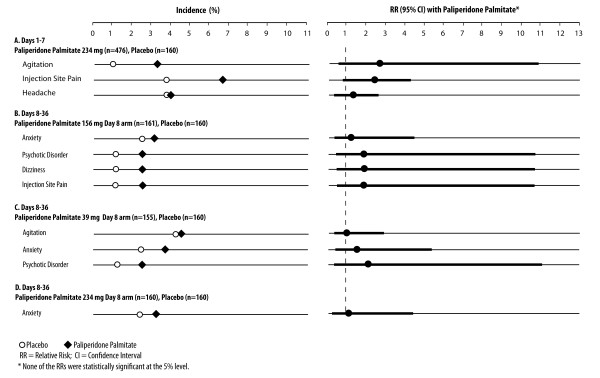
**Adverse Events in ≥2% of Paliperidone Palmitate and in a Higher Percentage of Paliperidone Palmitate than Placebo Subjects**. Adverse events meeting these criteria during Days 1 to 7 are shown in Panel A for subjects received paliperidone palmitate 234 mg Day 1 (rates and relative risks versus placebo with 95% CIs); none were statistically significant as determined by 95% CIs. Adverse events that met these criteria during Days 8 to 36 are shown in Panel B for the paliperidone palmitate 156 mg Day 8 group, Panel C for the 39 mg Day 8 group, and Panel D for the 234 mg Day 8 group. None were statistically significant as determined by 95% CIs.

Adverse events reported in ≥2% of paliperidone palmitate treated subjects and in a greater proportion of paliperidone palmitate than placebo-treated subjects were agitation (3.2% vs. 1.2%; RR 2.52 [95% CI 0.583, 10.904]), headache (4.0% vs. 3.8%; RR 1.06 [95% CI 0.433, 2.619]), and injection site pain (6.7% and 3.8%; RR 1.79 [95% CI 0.764, 4.208]) (Figure 4A). The RRs were not statistically significant as determined by 95% CIs.

The incidence of any EPS-related event reports during Days 1 to 7 was 3.6% (17/476) in the paliperidone palmitate 234 mg group and 3.1% (5/160) in the placebo group. The use of anti-EPS medications was 5.5% (26/476) and 7.5% (12/160), respectively.

Metabolic and potentially prolactin-related events were reported in < 2% of those administered paliperidone palmitate 234 mg or placebo on Day 1.

#### Days 8 to 36

The adverse event rate during the month following the Day 8 injection was 38.5% (62/161) in the paliperidone palmitate 156 mg Day 8 group and 41.3% (66/160) in the placebo group. Rates in the other paliperidone palmitate dose groups were 36.8% (57/155) with 39 mg Day 8, and 41.3% (66/160) with 234 mg Day 8.

A total of 39 subjects reported adverse events that were rated as serious during Days 8 to 36: 29 paliperidone palmitate subjects (6.1%) and 10 placebo subjects (6.3%). The serious adverse events reported in the placebo arm were: acute psychosis (1 subject); persecutory type delusional disorder (1 subject); psychotic disorder (2 subjects); schizophrenia (5 subjects); electrocardiogram change (1 subject); and non-cardiac chest pain (1 subject). Serious adverse events reported in those receiving the recommended Day 8 dose of 156 mg were: anxiety (1 subject); psychotic disorder (4 subjects); schizophrenia, paranoid type (1 subject); and schizophrenia (4 subjects).

Serious adverse events reported in the 39 mg Day 8 arm during this period were: agitation (1 subject); depression (1 subject); auditory hallucination (1 subject); insomnia (1 subject); psychotic disorder (2 subjects); schizophrenia (5 subjects); suicidal ideation (3 subjects); diverticulitis (1 subject); and syncope (1 subject). Those reported in the 234 mg Day 8 arm were: anxiety (1 subject); depression (1 subject); psychotic disorder (1 subject); schizophrenia (4 subjects); and cerebrovascular accident (1 subject). Note that a given patient may have reported more than one serious adverse event.

In the paliperidone palmitate group receiving the recommended initiation dosing (234 mg Day 1/156 mg Day 8), the adverse events reported in ≥2% of this group and in a greater percentage of paliperidone palmitate than placebo subjects were anxiety (3.1% vs. 2.5%; RR 1.24 [95% CI 0.340, 4.542]), psychotic disorder (2.5% vs. 1.3%; RR 1.99 [95% CI 0.369, 10.699]), dizziness (2.5% vs. 1.3%; RR 1.99 [95% CI 0.369, 10.699]), and injection site pain (2.5% vs. 1.3%; RR 1.99 [95% CI 0.369, 10.699]) (Figure 4B). These RRs were not statistically significant, as determined by 95% CIs.

In the paliperidone palmitate 39 mg Day 8 arm (Figure 4C), the adverse events reported in ≥2% of this group and in a greater percentage of paliperidone palmitate than placebo subjects were agitation (4.5% vs. 4.4%; RR 1.03 [95% CI 0.371, 2.874]), anxiety (3.9% vs. 2.5%; RR 1.55 [95% CI 0.446, 5.381]), and psychotic disorder (2.6% vs. 1.3%; RR 2.07 [95% CI 0.384, 11.110]). In the 234 mg Day 8 group (Figure 4D), the only adverse event meeting the criteria was anxiety (3.1% vs. 2.5%, RR 1.25 [95% CI 0.342, 4.570]).

The incidence of any EPS-related events during Days 8 to 36 were 3.7% (6/161) in the paliperidone palmitate 156 mg Day 8 arm and 4.4% (7/160) in the placebo arm (RR 0.8518; 95% CI 0.293, 2.48). Specific extrapyramidal symptoms were reported in < 2% of subjects in any treatment arm, with the exception of akathisia, reported in 2.5% (4/160) of the paliperidone palmitate 234 mg Day 8 arm and 3.1% (5/160) of the placebo arm (RR 0.800, 95% CI 0.219, 2.925).

The use of anti-EPS medications during Days 8 to 36 was 9.0% (14/155), 9.9% (16/161), and 6.3% (10/160) in those receiving 39, 156, and 234 mg on Day 8, respectively. The rate was 6.3% (10/160) in the placebo group.

Metabolic events and potentially prolactin-related events were reported in < 2% of subjects in each paliperidone palmitate arm and the placebo arm.

## Discussion

Two common clinical questions regarding the initiation dosing of paliperidone palmitate, specifically the time to onset of efficacy and the associated tolerability, were addressed in these post-hoc analyses of a large, double-blind, placebo-controlled trial. The question of when clinicians and patients can anticipate an improvement in symptoms is integral to clinical decision-making, particularly when managing a symptomatic patient with schizophrenia and planning a treatment strategy. While some clinicians may prefer to initiate paliperidone palmitate at a lower than the recommended initiation regimen due to tolerability concerns, previously published data suggest this may result in sub-therapeutic plasma levels and poor longer-term clinical response in some patients [9,11]. Thus, data were presented in this report for the early days and weeks following the initiation regimen to examine the efficacy and tolerability of the recommended initiation doses for paliperidone palmitate.

Findings showed significantly greater symptom improvement by Day 8 with paliperidone palmitate (234 mg on Day 1) compared to placebo, without oral antipsychotic supplementation, with this effect maintained after the 156 mg injections through Day 64, as well as at study endpoint [14]. When looking across the treatment arms, a trend towards a dose-dependent response was observed during the first 36 days of this study, again consistent with the data reported through study endpoint [14]. Also of note, the effect size vs. placebo for PANSS data illustrate an increasing improvement over time with the 156 mg dose (0.30, 0.43, 0.42, and 0.49 at Days 22, 36, 64, and endpoint, respectively), and the 234 mg dose (0.41, 0.40, 0.48, and 0.55, respectively). The 39 mg arm had lower and relatively constant effect sizes from Day 22 through endpoint (0.27, 0.28, 0.26, and 0.28, respectively). The early reduction in mean PANSS score shown here is supported by that from a non-inferiority trial [15], where PANSS improvement was similar at the Day 4 timepoint for subjects receiving an initial injection of paliperidone palmitate at 234 mg compared to oral risperidone given at 1 to 6 mg per day.

The clinical improvement observed with the initiation doses of paliperidone palmitate in this study is supported by the attainment of therapeutic serum concentrations of paliperidone reported in clinical and pharmacokinetic modeling analyses [7,8,14]. Following a single intramuscular dose, the release of paliperidone into the systemic circulation occurs as early as Day 1, with a gradual rise to reach maximum plasma concentrations at a median of 13 days [6]. The two initial doses of paliperidone palmitate (234 mg Day 1/156 mg Day 8) into the deltoid help attain therapeutic concentrations rapidly, with the AUC profiles being dose proportional over the 39 to 234 mg dose range [6]. In studies that used lower doses of paliperidone palmitate and initiation dose administration into the gluteal muscle, an onset of efficacy by Day 8 was not consistently observed [9,11].

With respect to tolerability concerns with the recommended paliperidone palmitate initiation dosing, this study did not reveal unexpected adverse events or high rates of specific adverse events in the first week or subsequent month after the initiation injections. In addition, overall treatment discontinuations and discontinuations due to adverse events were generally low during this time. However, these are data from a single clinical study. Further, the relative risk analysis requires comment. This analysis was undertaken with the intent of providing a useful way identifying adverse events that may be more likely to occur with active treatment as compared with placebo. Findings were that events such as agitation, anxiety, dizziness, headache, injection site pain, and psychotic disorder had a relative risk ranging from approximately 1.1 to 2.5 during the first month of treatment. Although these relative risks were not statistically significant, as determined by the 95% CIs, they may be clinically relevant providing useful information for clinicians to consider when initiating treatment with paliperidone palmitate. Additionally, it must be noted that the analysis of this relatively small database is not sufficient to identify rare treatment-related events.

Extrapyramidal symptoms such as parkinsonism, akathisia, dyskinesia, and dystonia are also an area of concern with respect to the tolerability of an antipsychotic regimen. Substantial literature supports that the incidence of these events as well as the time of onset differ substantially [16,17]. In terms of onset, dystonic reactions and akathisia generally occur within the first few hours to days of treatment while parkinsonism occurs within the first few weeks and tardive dyskinesia or dystonia generally appearing after months or years of treatment [16,17]. Broadly speaking the risk for extrapyramidal symptoms is generally considered to be lower with atypical compared with typical antipsychotics--however, the risk for these events varies among the agents in each class. Within the atypical class of agents the risk for extrapyramidal events is often dose-related [16]. In this analysis, the incidence of extrapyramidal symptoms was less than 2%, with akathisia being the only extrapyramidal symptom having an incidence of > 2% (2.5%) during Days 8 to 36 at the highest dose of paliperidone palmitate (234 mg).

One must also consider that this study was not designed to assess onset of efficacy or the tolerability associated with the initiation regimen. Therefore, these findings are somewhat limited by the timepoints that were assessed (i.e., Days 4, 8, 22, 36) and data collected at these visits. For example, more timepoints would be valuable to assess onset. It should also be noted that while commonly used criteria were applied to define onset as well as response, other criteria could result in different outcomes. Further, these criteria were applied to a population of subjects enrolled in a large double-blind clinical trial and these findings may not generalize to patient populations with different characteristics. Also, the results presented here are population-based data that do not fully address the heterogeneity that is associated with individual treatment response. That is, mean responses from a population address probabilities of clinical response but do not predict the response for a particular patient. Finally, it should be pointed out that there was a substantial placebo response observed in this trial. This is not uncommon in studies of patients with schizophrenia and, nevertheless, the effect size data for paliperidone palmitate compared to placebo suggests a clinically meaningful dose- and time-dependent treatment effect in this population.

## Conclusions

In this study, the initiation regimen of paliperidone palmitate of 234 mg on Day 1 and 156 mg on Day 8 was associated with a significant improvement in symptoms by Day 8 that continued at the subsequent Day 22 and Day 36 timepoints among subjects with symptomatic schizophrenia. There was a trend towards a dose-dependent response observed across the dosage groups. There were no unusual or unexpected tolerability findings noted during either the first week or month following paliperidone palmitate treatment initiation.

## Competing interests

The authors of this manuscript: Drs. Alphs, Bossie, Fu, and Sliwa are employees of Ortho-McNeil Janssen Scientific Affairs, LLC. The author Dr. Ma is an employee of Johnson & Johnson Pharmaceutical Research and Development, LLC.

## Authors' contributions

LA, CB, and JKS participated in the design of this analysis. YM performed the statistical analyses for this manuscript. All authors (LA, CB, JKS, YM, and DF) developed the draft of the manuscript and participated in its subsequent revisions. All authors (LA, CB, JKS, YM, and DF) read and approved the final manuscript.

## Endnote

^a.^ The dosing used in this clinical study aligns with the recommended initiation regimen of paliperidone palmitate (i.e., 234 mg on Day 1, 156 mg on Day 8); however, the dosage regimen recommends that these injections are both given in the deltoid muscle, with gluteal muscle injections being an option after the Day 8 dose [6].

## Pre-publication history

The pre-publication history for this paper can be accessed here:

http://www.biomedcentral.com/1471-244X/11/79/prepub
